# Two cases of severe incision infection after cesarean section: a case report

**DOI:** 10.3389/fmed.2026.1848627

**Published:** 2026-06-30

**Authors:** Wenzhe Zhou, Hongmei Qu

**Affiliations:** 1 Department of Obstetrics and Gynecology, The Affiliated Yantai Yuhuangding Hospital of Qingdao University, Yantai, China; 2 Shandong Provincial Maternal and Child Health Care Hospital Affiliated to Qingdao University, Jinan, China

**Keywords:** cause, cesarean section, incision, preventive measures, severe infection

## Abstract

Severe infection at the incision site after cesarean section is a rare occurrence. This article presents two cases of severe postoperative infection following cesarean section to explore the mechanisms, treatment, and prevention, while also investigating potential predisposing factors through the patients’ unique medical histories.

## Introduction

The probability of severe incision infection after cesarean section is very low, and there are few reports on this issue at present. Usually, its occurrence has individual characteristics. This article presents two cases of severe incision infection following cesarean section. Both patients have unique medical histories. Through these cases, we discuss the possible causes of incision infection, the selection of antibiotics, and preventive measures, aiming to draw greater attention from colleagues to this issue, implement reasonable and effective interventions to reduce the occurrence of such cases, and emphasize the importance of identifying high-risk patient characteristics.

## Case presentation

### Case report 1

The patient was a 24-year-old woman at 27 weeks of gestation. She was admitted to our hospital on November 11, 2024, because of abdominal contractions that had lasted for 4 days.

She had regular prenatal check-ups during pregnancy. Four days before admission, she received atosiban at a local hospital for irregular abdominal pain and was discharged after improvement. The pain worsened, leading to admission.

The patient had undergone cervical conization and laparoscopic cervical cerclage before pregnancy, complicated by postoperative fever and impaired healing of the laparoscopic incisions. She also had a history of 2 s-trimester spontaneous abortions and one first-trimester missed abortion.

Physical examination on admission showed: temperature 36.6 °C, pulse 90/min, respiration 18/min, blood pressure 119/80 mmHg, height 158 cm, weight 71.5 kg. The uterine fundus was two fingerbreadths above the umbilicus. Irregular uterine contractions were palpable with good relaxation between contractions, and there was no uterine tenderness.

After admission, intravenous atosiban and intramuscular dexamethasone were given. Ultrasound examination suggested a closed cervix of 3.7 cm in length, and a hyperechoic suture line was visible 3.0 cm from the external os.

On the afternoon of admission, the patient had vaginal fluid leakage suggestive of premature rupture of membranes. Vaginal secretions were sent for bacterial culture and sensitivity. An urgent blood test showed a white blood cell count of 21.28 × 10^9^/L and neutrophils 77.2%. Cefazolin 1 g was given intravenously every 8 h. The next day, repeat blood tests showed a white blood cell count of 19.47 × 10^9^/L, neutrophils 84.8%, and high-sensitivity C-reactive protein (hs-CRP) 28.83 mg/L. Given the rising infection markers, emergency lower segment cesarean section was performed for suspected intrauterine infection.

Intraoperatively, the myometrium was thick and fragile. A male infant weighing 1,100 g was delivered in the LOA position. Apgar scores were 6 at 1 min (heart rate 2, one point each for breathing, color, reflex, and muscle tone) and 8 at 5 min (one point deducted each for breathing and muscle tone). The amniotic fluid was clear and was sent for culture; the placenta was sent for pathological examination. The cervical cerclage suture was palpable near the sacral ligament on the posterior uterine wall, surrounded by adhesions.

On the day of surgery, antibiotics were upgraded to piperacillin-tazobactam 4.5 g q8h.

*Postoperative day 1*: The patient developed fever with chills at night; blood culture was sent.

*Postoperative day 2*: The temperature peaked at 39.0 °C. Antibiotics were upgraded to meropenem.

*Postoperative day 3*: The white blood cell count rose to 34.77 × 10^9^/L, neutrophils 85.7%, and hs-CRP 181.18 mg/L. Consultations with hematology and infectious disease were obtained. Doxycycline was added. CT showed no abnormalities.

*Postoperative day 5*: The abdominal incision became red and swollen, with progressive ulceration. Regular debridement and dressing changes were performed, but the ulcer continued to enlarge, and nightly fever persisted at 39.0 °C. Infectious disease consultation led to changing meropenem back to piperacillin-tazobactam and adding linezolid. No improvement was observed.

*Postoperative day 7*: Remittent fever persisted; the anti-infection regimen was temporarily unchanged.

*Postoperative day 9*: A multidisciplinary consultation (infectious disease, rheumatology, critical care, hematology) was held. Bone marrow aspiration and immune workup were performed; all results were negative.

*Postoperative day 10*: Pharmacy consultation was obtained. Necrotic tissue from the incision was sent for fungal and bacterial cultures. Antibiotics were changed to imipenem and fluconazole.

*Postoperative day 11*: The patient‘s temperature began to stabilize.

*Postoperative day 13*: Imipenem was de-escalated to piperacillin-tazobactam. Fluconazole and doxycycline were stopped, and linezolid was switched to oral administration.

*Postoperative day 16*: All antibiotics were discontinued.

*Postoperative day 30*: The patient was discharged.

*Six-month follow-up*: Complete wound healing.

**Figure 1:**
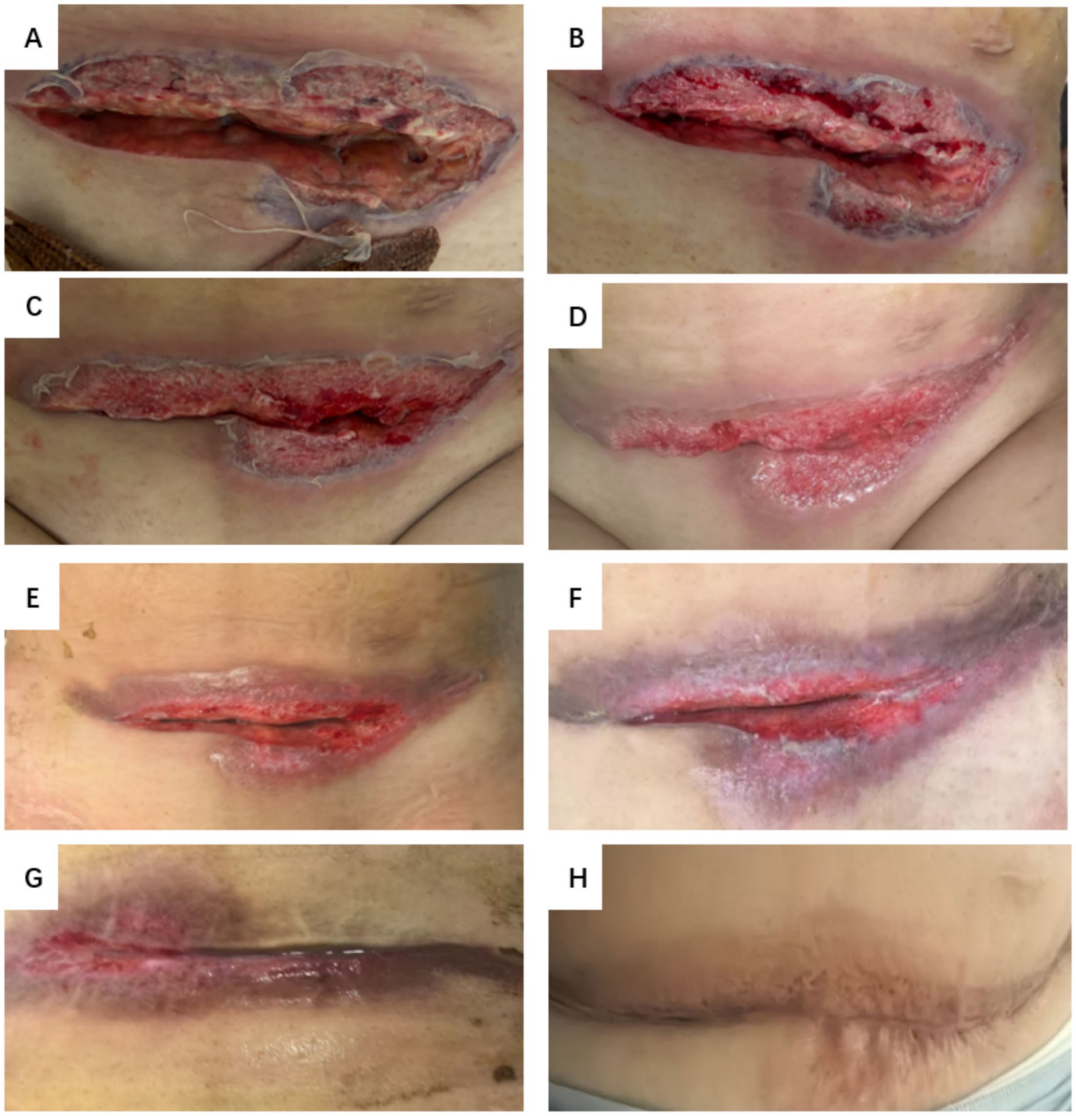
Case 1 wound evolution. **(A)** Day 5: skin ulceration around the incision; regular debridement and dressing changes, but progression. **(B)** Day 9: ulceration worsened; multidisciplinary consult, bone marrow biopsy (normal). **(C)** Day 11: wound ulcer begins to heal; necrotic tissue sent for culture on day 10, antibiotics upgraded to imipenem and fluconazole, fever decreases. **(D)** Day 13: fresh granulation tissue; de-escalation to piperacillin-tazobactam, stop fluconazole/doxycycline, linezolid oral. **(E)** Day 18: further healing; off antibiotics since day 16. **(F)** Day 28: further healing; discharged day 30. **(G)** Day 42: almost healed. **(H)** 6 months: completely healed.

*Microbiological and pathological results*: Two vaginal secretion cultures, one amniotic fluid culture, two blood cultures (one long-term), and ulcer tissue culture were all negative. Placental pathology showed chorioamnionitis stage II, grade III (the wound evolution is shown in Figure 1). Patient characteristics and treatment timeline are summarized in Tables 1, 2, respectively.

**Table 1:** Admission summary of Case 1 and Case 2.

Category	Case 1	Case 2
Patient demographics	Female, 24 years old	Female, 33 years
Presenting symptoms	27 weeks, abdominal contractions for 4 days; atosiban at local hospital, discharged, then pain worsened; admitted Nov 11, 2024	39 weeks, bloody show 2 days, lower abdominal pain ½ day. Normal prenatal care, no special medications.
Past medical/surgical history	Cervical conization and laparoscopic cervical cerclage before pregnancy	Blood transfusion for anemia 10 years ago. No underlying diseases, surgery, or trauma.
Obstetric history	G4P0; two second-trimester spontaneous abortions; one first-trimester missed abortion	G2P1, one vaginal delivery at age 25.
Physical examination	T 36.6 °C, P 90/min, R 18/min, BP 119/80 mmHg; 158 cm, 71.5 kg; fundus +2 fingerbreadths; irregular contractions, good relaxation, no tenderness	T 36.3°C, P 90/min, R 20/min, BP 124/79 mmHg, fundal height 34 cm, LOA, FHR 145 bpm, cervical dilation 2 cm, membranes intact.
Laboratory findings (admission)	WBC 21.28 × 10^9^/L, neutrophils 77.2% (after PROM)	Hb 75 g/L, WBC 11.5 × 10^9^/L, PLT 49 × 10^9^/L.
Imaging findings	Ultrasound: cervical length 3.7 cm, closed; suture echo 3.0 cm from external os	Singleton, third trimester.
Admission diagnoses	Threatened miscarriage; 27 weeks G4P0; cervical insufficiency; status post cervical cerclage and conization; history of adverse pregnancy outcomes	39 weeks gestation, thrombocytopenia (cause undetermined), moderate anemia.

**Table 2:** Treatment timeline, clinical outcome and follow-up (Case 1).

Time point	Event / intervention	Clinical findings / outcome
Admission day (Nov 11)	IV atosiban, IM dexamethasone; later PROM, cefazolin	Ultrasound: cervix closed, 3.7 cm; WBC 21.28
Next day	Emergency cesarean section due to rising infection markers; antibiotics upgraded to piperacillin-tazobactam	Male infant 1,100 g, Apgar 6 → 8; WBC 19.47, CRP 28.83
Post-op days 1–3	Fever to 39 °C, antibiotics upgraded to meropenem, doxycycline added	WBC 34.77, CRP 181.18; CT normal
Post-op day 5	Abdominal ulceration; regular debridement and dressing changes; antibiotics changed to piperacillin-tazobactam + linezolid	Ulcer progressed; no improvement
Post-op days 9–10	Multidisciplinary consultation; bone marrow aspiration and immune tests (normal); changed to imipenem + fluconazole	Temperature stabilized on post-op day 11
Post-op days 13–16	De-escalation to piperacillin-tazobactam; stopped fluconazole and doxycycline; linezolid switched to oral; all medications stopped by post-op day 16	Fresh granulation tissue appeared; wound began to heal
Post-op day 30	Discharged	Follow-up at day 42: wound nearly completely healed
Microbiology and pathology	2 vaginal secretion cultures, 1 amniotic fluid culture, 2 blood cultures (1 long-term), placental pathology, ulcer tissue culture for pyogenic bacteria	All negative except placental pathology: chorioamnionitis stage II, grade III

### Case report 2

The patient was a 33-year-old woman at 39 weeks of gestation who was admitted because of bloody show for more than 2 days and lower abdominal pain for half a day. The pregnancy was spontaneously conceived, and she had regular prenatal check-ups; blood glucose and blood pressure were normal throughout pregnancy, and she took no special oral medications. Her past medical history included a blood transfusion for anemia 10 years before admission; otherwise she had no underlying diseases, infectious diseases, or history of surgery or trauma. Her obstetric history included one previous full-term vaginal delivery at age 25 (uncomplicated), and the current pregnancy was her second (G2P1). On admission, physical examination showed temperature 36.3 °C, pulse 90/min, respiration 20/min, blood pressure 124/79 mmHg, height 154 cm, weight 61 kg, fundal height 34 cm, abdominal circumference 96 cm, fetal position LOA, fetal heart rate 145 bpm, cervical dilation 2 cm, and membranes intact. Laboratory findings revealed hemoglobin 75 g/L, white blood cell count 11.5 × 10^9^/L, and platelet count 49 × 10^9^/L. Ultrasound showed a singleton pregnancy in the third trimester. Admission diagnoses were 39 weeks gestation, G2P1, LOA, thrombocytopenia (cause to be determined), and moderate anemia. On the day after admission, she underwent lower segment cesarean section with ligation of the ascending branch of the uterine artery.

*Postoperative day 2*: Blood tests showed white blood cells 11.47 × 10^9^/L, hemoglobin 81 g/L, and platelets 4 × 10^9^/L. Hematology consultation was obtained. Treatment included intravenous hemostatic agents, platelet transfusion, and subcutaneous recombinant human thrombopoietin (rhTPO).

*Postoperative day 3*: The patient developed palpitations, chest tightness, and shortness of breath. Blood tests showed white blood cells 2.56 × 10^9^/L, hemoglobin 91 g/L, platelets 10 × 10^9^/L, and BNP 17725 pg./mL. Heart failure and pulmonary infection were suspected. Meropenem was started, recombinant human granulocyte colony-stimulating factor (G-CSF, 200 mcg) was given, and 2 units of red blood cells were transfused.

*Postoperative day 5*: Peritoneal drainage fluid culture was positive for *Streptococcus pyogenes*, and sputum culture was positive for *Acinetobacter baumannii*. Antibiotics were adjusted to a carbapenem, vancomycin, caspofungin, and minocycline. Comprehensive supportive measures were initiated, including intravenous immunoglobulin (IVIG), coagulation factors, continuous hemofiltration, and therapeutic plasma exchange. Subsequently, the patient‘s renal and respiratory functions gradually recovered.

*Postoperative day 17*: The skin around the abdominal incision became blackened, with redness and swelling.

*Postoperative day 26*: A small amount of exudate appeared at the incision site. Local skin necrosis, subcutaneous abscess formation, and pitting edema were noted.

*Postoperative day 43*: A large area of skin and subcutaneous adipose tissue was missing at the incision site, with sinus openings and fluid exudation. Both necrotic tissue and fresh granulation tissue were visible. Debridement was performed to remove necrotic tissue. Daily wound care was carried out using Aikexin dressing combined with nano-silver gel.

**Figure 2:**
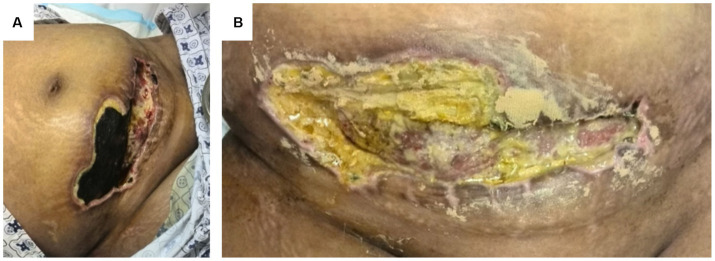
Case 2 wound appearance. **(A)** Postoperative day 37: Periwound skin necrosis, subcutaneous abscess formation, and pitting edema of the surrounding abdominal wall. **(B)** Postoperative day 40: Extensive loss of skin and subcutaneous fat, with both necrotic tissue and fresh granulation tissue present in the wound.

*Postoperative day 49*: After the patient‘s vital signs stabilized, she was transferred to the Burn Unit. There, wound management consisted of weekly surgical debridement and negative pressure wound therapy (NPWT) for a total of 21 sessions, followed by regular dressing changes until complete healing was achieved (the wound appearance is shown in Figure 2). Patient characteristics and treatment timeline are summarized in Tables 1, 3, respectively.

**Table 3:** Treatment timeline, clinical outcome and follow-up (Case 2).

Time point	Event / intervention	Clinical findings / outcome
Admission day 2 (Feb 9)	Elective cesarean section + uterine artery ligation	–
Postoperative day 2	WBC 11.47 × 10^9^/L, HGB 81 g/L, PLT 4 × 10^9^/L	Hemostatic agent, platelet transfusion, rhTPO (15,000 IU subcutaneously once daily)
Postoperative day 3	Palpitations, dyspnea; WBC 2.56 × 10^9^/L, HGB 91 g/L, PLT 10 × 10^9^/L, albumin 25.7 g/L, CRP 428 mg/L, BNP 17725 pg/ml	Meropenem for anti-infection, G-CSF (200 mcg) to raise WBC, RBC transfusion (2 U)
Postoperative day 5	Peritoneal drainage fluid culture: *Streptococcus pyogenes* positive; sputum culture: *Acinetobacter baumannii* positive	Antibiotics changed to carbapenem, vancomycin, caspofungin, minocycline. Added IVIG, coagulation factors, continuous hemofiltration, plasma exchange. Patient‘s renal and respiratory functions gradually recovered;
Postoperative day 17	Incision skin turned black, surrounding redness and swelling	Debridement; Biafine topical once daily, nano-silver gel topical once daily
Postoperative day 26	Small amount of exudate at incision; local skin necrosis, subcutaneous abscess, pitting edema	Sutures removed, local wound care, continued active anti-infection therapy
Postoperative day 43	Large area of skin and subcutaneous fat loss, sinus openings with fluid exudation; mixed necrotic and fresh granulation tissue	Debridement performed. Daily wound care with Aikexin dressing combined with nano-silver gel topical application.
Postoperative day 49	Transferred to Burn Unit; wound debridement + VAC negative pressure therapy (21 sessions), then regular dressing changes until complete healing	Wound healed

## Discussion

Severe infection at the incision site after cesarean section is uncommon. The causes are often difficult to determine completely. According to the literature (1, 2), prolonged labor, chorioamnionitis, long duration of premature rupture of membranes, repeated vaginal examinations, decreased hemoglobin level, obesity, longitudinal skin incision, and general anesthesia are positively correlated with the incidence of postoperative incision infection. In Case 1, the patient had premature rupture of membranes at admission, and placental pathology confirmed chorioamnionitis (stage II, grade III). Infected amniotic fluid or fetal membranes can directly contaminate the incision during surgery, leading to bacterial colonization and postoperative wound ulceration and necrosis (3). The patient’s body mass index was 28.6 kg/m^2^ (overweight). In obese patients, adipose tissue has poor circulation, increasing the risk of fat liquefaction and fat necrosis. The patient also had a history of poor wound healing after a previous laparoscopic procedure. Recurrent miscarriages may also suggest immune dysregulation. Some immune-related diseases, such as pyoderma gangrenosum (PG), can also cause postoperative wound necrosis and ulceration. In Case 2, the patient had moderate anemia (hemoglobin 75 g/L at admission) and thrombocytopenia (platelet count 49 × 10^9^/L). Anemia causes tissue hypoxia, impairs wound healing, and reduces immunity, aggravating postoperative infection (4). Platelets play a critical role in wound healing and tissue repair. They recognize, bind, and manipulate extracellular structures, detect pathogens and tissue damage, and release multiple mediators that coordinate platelets and other cells in tissue repair (5). Thrombocytopenia also affects postoperative wound healing.

In Case 1, the patient’s blood test results showed elevated white blood cell count, percentage of neutrophils, and C-reactive protein, all of which suggested that the patient might have an infection. However, the results of bacterial cultures of vaginal secretions, blood, and necrotic tissue from the surgical incision were all negative. This might be due to the use of antibiotics affecting the results of the bacterial culture, but it is also possible that there are other reasons for this. This patient has a history of two spontaneous abortions and one missed abortion. Moreover, she underwent laparoscopic surgery 1 year ago, and the surgical wound did not heal properly. This suggests that the patient may have underlying immune dysregulation. van Donkelaar et al. (6) reported a case of severe infection after cesarean section. Multiple cultures (blood, wound, and vaginal) were negative. On the 5th postpartum day, the cesarean section wound showed purulent discharge and dehiscence. Antibiotic treatment was ineffective, and the final diagnosis was Pyoderma Gangrenosum (PG). The clinical symptoms improved rapidly after receiving high-dose systemic corticosteroid treatment (prednisolone 1 mg/kg). PG is a rare neutrophilic dermatosis, closely related to auto-inflammatory diseases. The diagnostic criteria for PG require the fulfillment of two main criteria and two secondary criteria, as shown in Table 4. According to the diagnostic criteria, this case does not seem to be typical. Corticosteroids were not administered due to concern for occult infection. Skin biopsy was not performed. Therefore, PG cannot be confirmed, and this diagnostic uncertainty is a major limitation of the present report. Case 2 had a confirmed infectious etiology. Peritoneal drainage fluid culture was positive for *Streptococcus pyogenes* (Group A Streptococcus). The wound necrosis was caused by GAS toxic shock syndrome.

**Table 4:** Proposed diagnostic criteria for pyoderma gangrenosum.

Diagnostic criteria: two major and two out of four minor	
Major criteria	Minor criteria
1. Rapid progression of a painful, necrolytic, cutaneous ulcer with an irregular, violaceous border	1. History suggestive of pathergy or clinical finding of cribriform scarring
2. Exclusion of other causes of cutaenous ulceration	2. Systemic diseases associated with pyoderma gangrenosum
	3. Compatible histopathological findings
	4. Response to treatement

Severe wound breakdown after cesarean section can result from several mechanisms. In Case 2, *Streptococcus pyogenes* (Group A Streptococcus) produced exotoxins and proteases that degrade extracellular matrix, induce tissue necrosis, and trigger a cytokine storm. This leads to microvascular thrombosis, DIC, and ischemic necrosis of the surgical site (7). In Case 1, the pathophysiology remains unclear. The most likely mechanism is occult bacterial infection: prolonged antibiotics may have reduced bacterial load below culture thresholds, but residual bacteria could still cause necrosis. Alternatively, the clinical presentation resembles pyoderma gangrenosum (PG), where neutrophil-driven sterile inflammation and pathergy lead to tissue necrosis independent of infection.

Several alternative causes of postoperative wound ulceration were considered. In Case 1, Necrotizing fasciitis was excluded by the absence of systemic toxicity, negative CT for fascial gas or thickening, intact fascial planes at debridement, and a LRINEC score below 6. Atypical mycobacterial infection typically has an indolent, subacute course (weeks to months) and lacks marked systemic inflammation, which contrasts with the acute onset (postoperative day 5) and high fever/leukocytosis/CRP in this case. Deep fungal infection is unlikely in an immunocompetent host without risk factors. Drug-associated skin necrosis was considered but is unlikely given that none of the administered drugs have a known causal link with incisional necrosis and no temporal association was identified. Vasculitis and autoimmune neutrophilic dermatoses (e.g., Sweet syndrome) were considered but are not supported by the absence of systemic symptoms, negative autoantibodies (ANA, ANCA), and lack of histopathology. Culture-negative bacterial infection is the most likely cause, supported by fever, leukocytosis, elevated CRP, and eventual response to imipenem. Negative cultures are explained by prior antibiotic exposure. In addition, pyoderma gangrenosum (PG) remains a differential diagnosis that cannot be excluded without histopathological examination. In Case 2, at admission, the patient had moderate thrombocytopenia (platelet count 49 × 10^9^/L). The differential diagnosis included gestational thrombocytopenia, immune thrombocytopenia (ITP), HELLP syndrome, and early consumptive coagulopathy. HELLP syndrome was excluded because the patient had normal blood pressure during pregnancy, no proteinuria, and normal liver function before admission. ITP was considered but not diagnosed, as the patient had no history of thrombocytopenia before pregnancy and the platelet count recovered after infection control. On postoperative day 2, the platelet count dropped sharply from 49 × 10^9^/L to a nadir of 4 × 10^9^/L, in the setting of GAS sepsis. Subsequent coagulation studies showed abnormalities (PT 16.9 s, APTT 68.2 s, D-dimer 2.10 mg/L, fibrinogen 1.70 g/L). Therefore, the severe thrombocytopenia was attributed to sepsis-induced DIC with consumptive coagulopathy, rather than ITP or HELLP syndrome. Furthermore, because platelet autoantibodies, serum folate/B12, and bone marrow examination were not performed, the cause of the antenatal thrombocytopenia (platelet count 49 × 10^9^/L) remains undetermined, which is a limitation of this report.

In Case 1, all cultures (vaginal secretions, amniotic fluid, blood, and necrotic tissue from the ulcer edge) were negative. The patient received intravenous cefazolin before vaginal and blood sampling on admission. By postoperative day 10, when necrotic tissue was sent for culture, she had already been treated sequentially with cefazolin, piperacillin-tazobactam, meropenem, and linezolid. This extensive prior antibiotic exposure likely suppressed bacterial growth, reducing the bacterial load below detectable thresholds. Furthermore, conventional cultures cannot reliably detect fastidious organisms (e.g., Ureaplasma, Mycoplasma), anaerobes, atypical mycobacteria, or fungi without special media or prolonged incubation; sensitivity in patients already on antibiotics is reported to be only 40% (8). Therefore, negative cultures do not exclude infection. Unlike conventional culture methods, molecular diagnostic techniques such as PCR and metagenomic next-generation sequencing (mNGS) do not require viable bacteria for detection. They directly identify pathogen-specific nucleic acids, a feature that makes them robust even in patients who have received antibiotics. These methods also significantly shorten turnaround time, meeting the diagnostic needs of critically ill patients. More importantly, they can detect fastidious or unculturable microorganisms, including viruses, bacteria, fungi, and parasites, which is a major advantage in cases of rare, mixed, or unexplained infections (9). Given the diagnostic difficulties in Case 1, we recommend incorporating these molecular techniques into the diagnostic workup of future culture-negative infections.

How to choose antibiotics to prevent incision infection after cesarean section? We need to select antibiotics that are effective against Gram-positive bacteria, Gram-negative bacteria, and some anaerobic bacteria to prevent post-operative infections after a cesarean section (10). Cefazolin has significant advantages in both efficacy and cost-effectiveness. According to the ACOG Practice Bulletin (11), intravenous administration of 2 g of cefazolin within 1 h before surgery has a good effect in preventing postoperative infections. Meanwhile, Mei et al. (12) believe that cefazolin is the preferred antibiotic for preventing postoperative infections after cesarean section. However, for pregnant women with high-risk factors for infection, such as premature rupture of membranes, obesity, and postpartum hemorrhage, the use of antibiotics, including the dosage, administration route, and whether to combine them, still requires further research and exploration. For patients with chorioamnionitis, the selection of antibiotics should not only cover aerobic and anaerobic bacteria, but also take into account the possibility of genital tract mycoplasma infection. Therefore, Dr. Eunjung Jung and colleagues explored the use of a combined antibacterial therapy consisting of ceftriaxone, clarithromycin, and metronidazole for the treatment of chorioamnionitis (10). The advantage of this combination is that the antibacterial spectrum of the antibiotics covers both aerobic and anaerobic bacteria, as well as mycoplasmas, of the reproductive tract, and it is also effective in treating intra-amniotic sterile inflammation. The research conducted by Oh et al. (13) employed a combination of ceftriaxone, clarithromycin, and metronidazole as antibiotics to treat pregnant women with premature rupture of membranes and chorioamnionitis. The results indicated that even in the absence of detectable pathogenic microorganisms, antibiotics could be effective in treating patients with intra-amniotic inflammation. The above research results all suggest that in cases of preterm premature rupture of membranes, especially for patients with high-risk factors for chorioamnionitis, antibiotic treatment should involve a combined medication regimen effective against aerobic bacteria, anaerobic bacteria, and mycoplasma. In Case One, cefazolin was used preoperatively, and the antibiotic was upgraded to piperacillin sodium and tazobactam sodium postoperatively. Later, it was upgraded to meropenem, but the effect was unsatisfactory. It was not until imipenem and cilastatin were used that the body temperature returned to normal and the skin ulceration was controlled. Both meropenem and imipenem are carbapenem antibiotics with a broad antibacterial spectrum and strong antibacterial activity against Gram-negative, Gram-positive bacteria, and anaerobic bacteria. However, compared with meropenem, imipenem demonstrates stronger antibacterial activity against Gram-positive bacteria. After switching to imipenem on postoperative day 10, the patient became afebrile within 24 h. White blood cell count declined from 19.03 × 10^9^/L on day 10 to 11.03 × 10^9^/L on day 16, and neutrophil percentage decreased from 84.1 to 68.2% over the same period, with no concurrent changes in management. This temporal association suggests that the change to imipenem was associated with clinical and laboratory improvement, although causality cannot be proven. Case two is a severe sepsis caused by *Streptococcus pyogenes*. The choice of antibiotics should be based on the results of drug sensitivity tests.

Apart from the use of antibiotics, factors such as preoperative vaginal disinfection, screening and treatment of bacterial vaginosis, methods for removing the placenta during surgery, and changing gloves during the operation have all been proven to affect the occurrence of incision infections after cesarean section. Studies have shown that using povidone-iodine or chlorhexidine solution for vaginal disinfection before a cesarean section can reduce the number of bacteria in the vagina thereby avoiding infection of the endometrium and surgical wounds (14). The latest practice bulletin from ACOG on “Prevention of Infections after Gynecological Surgery” states that conducting a BV test (if present) before the surgery and treatment can reduce the risk of SSI in this population (15). A 2010 review demonstrated that manual placental extraction was associated with a significantly increased risk of endometritis compared with umbilical cord traction and fundal massage (16). The meta-analysis conducted by Narice et al. (17) indicates that replacing gloves after the placenta is delivered during cesarean section can significantly reduce the incidence of infectious wound complications.

## Conclusions and clinical implications

Severe post-cesarean wound ulceration is rare but challenging. The two cases provide practical clinical lessons.

First, microbiological samples should be collected before antibiotic administration. In Case 1, except for the vaginal secretion culture taken at the time of premature rupture of membranes, all other cultures were obtained after antibiotics had been started. This may have compromised culture accuracy, resulting in repeated negative results and preventing targeted antibiotic therapy.

Second, when infection is strongly suspected clinically but cultures are repeatedly negative, molecular diagnostic techniques (e.g., metagenomic next-generation sequencing, mNGS) should be considered to detect fastidious or suppressed pathogens. This was a limitation in Case 1 and an important lesson.

Third, timely, appropriate, and adequate antibiotic therapy is essential. In Case 1, after admission with premature rupture of membranes, the white blood cell count was 21.28 × 10^9^/L with 77.2% neutrophils, already indicating possible severe infection. However, only cefazolin 1 g q8h was given – the antibiotic class and spectrum were insufficient – and pregnancy was not terminated promptly. These factors may be closely related to the subsequent wound infection. In Case 2, the patient had thrombocytopenia and anemia, suggesting possible impaired immune function and wound healing capacity, placing her at higher risk of postoperative infection, yet only routine prophylactic antibiotics were administered.

Fourth, for progressive ulcers that do not respond to antibiotics, early skin biopsy from the ulcer edge is essential to exclude non-infectious causes such as pyoderma gangrenosum.

Fifth, individual patient characteristics must be considered. In Case 1, the patient had a history of recurrent miscarriages, which may indicate an underlying immune disorder; therefore, an immune workup should be performed before pregnancy. In addition, she had a history of poor wound healing after a previous laparoscopic procedure, suggesting a high risk of recurrent healing problems. For such patients, postoperative measures such as infrared therapy, topical application of rhubarb and mirabilite, and early wound disinfection and dressing changes should be initiated as soon as possible. In Case 2, the patient had thrombocytopenia, anemia, and obesity – all risk factors for infection. Obese patients are at greater risk of wound fat liquefaction. For such patients, adequate antibiotics should be given promptly, and wound care should begin early after surgery.

In addition, intraoperative aseptic principles must not be overlooked: preoperative vaginal disinfection, spontaneous placental delivery, glove change after placental delivery, and disinfection of the incision before skin closure are all effective measures to reduce postoperative wound infection.

## Data Availability

The original contributions presented in the study are included in the article/supplementary material, further inquiries can be directed to the corresponding author.
